# ARG1 is a potential prognostic marker in metastatic and recurrent endometrial cancer

**DOI:** 10.21203/rs.3.rs-2917380/v1

**Published:** 2023-07-18

**Authors:** Dinh Nam Tran, Valery Rozen, Mark I. Hunter, Tae Hoon Kim, Jae-Wook Jeong

**Affiliations:** University of Missouri School of Medicine; Michigan State University College of Human Medicine; University of Missouri School of Medicine; University of Missouri School of Medicine; University of Missouri School of Medicine

**Keywords:** ARG1, Endometrial Cancer, Metastasis, PTEN

## Abstract

Endometrial cancer (EC) is the most common gynecologic malignancy. While the majority of patients present with early-stage and low-grade EC and have an excellent prognosis, a subset has metastatic disease at presentation, or develops distant recurrence after initial treatment of the primary. However, the lack of prognostic biomarkers for metastatic EC is a critical barrier. Arginase 1 (ARG1) regulates the last step of the urea cycle, and an increase in ARG1 has been correlated as a poor prognostic factor in a variety of cancers. In the present study, ARG1 expression was evaluated as a potential prognostic marker for metastatic EC in endometrial hyperplasia and cancer of mice with *Pten* mutation as well as *Pten and Mig-6* double mutations. While *Pten* mutation in the uterus is not sufficient for distant metastasis, mice with concurrent ablation of *Mig-6* and *Pten* develop distant metastasis. Our immunostaining and RT-qPCR analysis revealed that the expression of ARG1 in early stage of EC as well as endometrial hyperplasia from mice deficient in *Mig-6* and *Pten* mutations significantly increased compared to *Pten* mutation in the uterus. The results suggest that a high level of ARG1 is associated with poor prognosis in association with EC of mouse.

## INTRODUCTION

Endometrial cancer (EC) is the most common malignancy of the female genital tract and fourth most common type of cancer affecting women in the United States [1]. According to the American Cancer Society, there will be ~ 66,200 new uterine cancer cases in the United States in 2023, with an annual mortality close to 13,030 [2]. Endometrioid endometrial cancer (EEC) is the most common histological type of EC, constituting 75–80% [3], and is mostly diagnosed at an early stage [4]. Prognosis for early-stage and low grade EEC is generally favorable: 5-year survival is 69–88% for FIGO (International Federation of Gynecology and Obstetrics) stage I–II disease [4]. However, despite successful surgery (hysterectomy) 10–15% of EEC tumors recur within 5 years with low survival rates [3].

At the time of diagnosis, stage is the most important variable for survival. Other valuable prognostic factors include age, nuclear grade, histologic type and grade, tumor size, and hormone receptor status. In most scenarios, staging guides the management of either surgical or non-surgical therapies. Current robotic surgery approaches including total hysterectomy with bilateral salpingo-oophorectomy is the mainstay of treatment. Most women with EC treated with surgery have 95% probability of survival at 5 years, as an estimated 55% of patients at the time of diagnosis have uterine-confined malignancy [5]. Early-stage (stage I and II) and low-grade EC present with a favorable prognosis, in comparison to patients with stage III or IV EC with poorer outcomes. Women with grade III disease and deep myometrial invasion are at risk for recurrence and metastatic disease [6]. The use of both chemotherapy and radiation has been associated with improved survival in individuals with advanced EC staging compared to either modality used alone [7], nonetheless despite several trials involving mTOR and PI3K inhibitors, there are not current approved targeted therapies due to infectiveness in EC treatment [8]. Median survival for metastatic EC is roughly 12 months, and despite the availability of several therapy modalities to treat this subset of patients, overall prognosis remains poor, and the incurable nature of the recurrent cancerlikelihood persist an impediment towards achieving remission [9]. If we could identify which patients are at highest risk of EEC recurrence, we would know who to offer adjuvant treatment or extensive surgical staging. But until we can identify prognostic biomarkers for EEC recurrence and metastasis, identifying these high-risk patients will likely remain beyond our capabilities.

*PTEN* is a negative regulator of PI3K signaling and is deficient (either completely lost or mutated) in > 50% of primary human EECs [10]. Furthermore, the PTEN/PI3K/AKT pathway is the most frequently altered signaling pathway in EEC [10]. AKT activation caused by loss of *PTEN* results in activating ESR1-dependent pathways vital to EEC tumorigenesis [11]. Most EECs manifest proliferating epithelial cells, increased AKT signaling and an association with unopposed estrogen (E2) exposure that plays a key role in EEC tumorigenesis.

On the other hand, progesterone (P4) antagonizes the growth-promoting properties of E2 in the uterus [12, 13]. Mitogen-inducible gene 6 (MIG-6; ERRFI1; RALT; gene 33) is a 50 kDa adaptor protein [14] and an immediate early-response gene inducible by various mitogens and commonly occurring chronic stress stimuli [14–16], Down-regulated expression of MIG-6 has been seen in human lung cancer [17], papillary thyroid cancer [18], and breast carcinoma [19–21], suggesting that MIG-6 has a human tumor suppressor role. We have identified MIG-6 as a tumor suppressor in human EEC [22–24] that mediates P4 signaling to inhibit E2 signaling in both the murine and human uterus [23, 25, 26], Ablating *Mig-6* in the mouse uterus leads to development of Complex Atypical endometrial Hyperplasia (CAH) and E2-induced EEC [23], Strikingly, overexpression of *Mig-6* also suppresses tumor development in *PTEN*-negative EEC [24], Importantly, *Pten* mutation is not sufficient for distant metastasis, but mice with concurrent ablation of *Mig-6* and *Pten* develop distant metastasis [27], Our mouse model is resembling distant metastasis EEC in women.

Based on a systematic review on EC prognostic markers, lack of discovery is one of the main causes for the current lack of research addressing the clinical gaps seen in EC [28], In the present study, we aim to provide clinically translatable molecular data addressing EC prognostic biomarkers. We identified *ARG1,* the gene coding the arginase 1 enzyme, as a potential EC prognostic biomarker. Arginase regulates the last step of the urea cycle, and an increase in this enzyme has been correlated as a poor prognostic factor in a variety of cancers including ovarian carcinoma, colorectal cancer, and neuroblastoma [29]. L-ornithine, one of the metabolites of L-arginine, can be furthered metabolized into polyamines required for DNA synthesis, serving a vital role in distinct cellular functions such as cell proliferation [30], Despite reported associations with other types of cancer, there are no current studies addressing the relationship between metastatic or recurrent EC and levels of ARG1. Therefore, the aim of this study was to evaluate the association and prognostic potential of ARG1 levels for EC using preclinical mouse models.

## Materials and Methods

### Animals and tissue collection

All animal experiments were approved by the University of Missouri Animal Care and Use Committee. Mice were housed and bred in a designated animal care facility at University of Missouri with controlled humidity and temperature conditions and a 12 h light/dark cycle. Mice of various genotypes were sacrificed at 2 weeks, 1 and 2 months of age (n = 5 per genotype). Uterine tissues were collected at the time of dissection and briefly fixed with 4% (vol/vol) paraformaldehyde for immunohistochemistry or snap frozen and stored at −80°C for RNA/protein extraction.

### Quantitative real-time PCR

Total RNA was extracted from the uterine tissues using the RNeasy total RNA isolation kit (Qiagen, Valencia, CA). NanoDrop was used to determine RNA purity and for an initial estimate of RNA concentration. 1 μg of RNA was used for reverse transcription with MMLV Reverse Transcriptase (Invitrogen Crop) according to the manufacturer’s instructions. mRNA expression levels of genes of interest were measured by real-time PCR TaqMan or SYBR green analysis using an Applied Biosystems StepOnePlus system (Applied Biosystems, Foster City, CA, USA). The mRNA quantities were normalized against the housekeeping gene, 60S ribosomal protein L7. Analysis of mRNA expression was first undertaken by the standard curve method, and results were corroborated by cycle threshold values assessing gene expression.

### Immunostaining

Immunohistochemistry analyses were performed as previously described ^40,41^. Dewaxed hydrated paraffin-embedded tissue sections were blocked with 10% normal goat serum in PBS (pH 7.5) and then incubated with primary antibody diluted in 10% normal goat serum in PBS (pH 7.5) overnight at 4°C (anti-ARG1 (1:2000 dilution; ab32037; Abeam). On next day, the sections were incubated with the appropriate species specific HRP-conjugated secondary antibody (2 μg/ml; Vector Laboratories) for one hour at room temperature. Immunoreactivity was detected using the Vectastain Elite DAB kit (Vector Laboratories). A semiquantitative grading system (H-score) was used to compare the immunohistochemical staining intensities as previously described ^42^. The overall score ranged from 0 to 300.

### Statistical analysis

To assess statistical significance of parametric data, the Student’s t test was used for data with only two groups. For data containing more than two groups, one way ANOVA was used, followed by Tukey’s post hoc test for multiple comparisons. All data are presented as means ± SEM. *p* < 0.05 was considered statistically significant. All statistical analyses were performed using the Prism9 package from GraphPad (San Diego, CA, USA).

## RESULTS

### The expression of ARG1 protein is significantly increased in EC from Pten ^d/d^ Mig-6^d/d^ mice compared to Pten^d/d^ mice.

**Figure 1:**
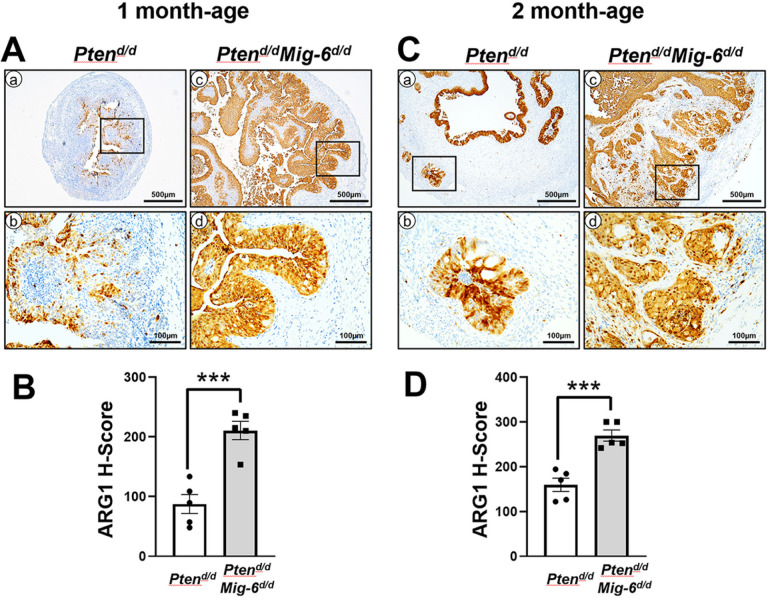
An increase in expression of ARG1 in *Pten*^*d/d*^*Mig-6*^*d/d*^ mice. Immunohistochemical analysis of ARG1 and quantification of ARG1 positive cells in the uteri of *Pten*^*d/d*^*, Pten*^*d/d*^*Mig-6*^d/d^at 1 month (**A**) and 2 months (**C**) of age. (**B**) and (**D**) Immunohistochemical H-score of (**A**) and (**C**). The results represent the mean ± SEM. ***p < 0.001.

Previously, we found that *Mig-6* ablation in *Pgr*^*cre/+*^*Pten*^*f/f*^ *(Pten*^*d/d*^) mice dramatically accelerated the development of EC compared with the single ablation of *Pten* [31]. While *Pten*^*d/d*^ mice showed endometrial hyperplasia in the uteri at one month of age and then developed EC with invasion into the myometrium occurring by 2 months [32], *Pgr*^*cre/+*^*Pten*^*f/f*^*Mig-6*^*f/f*^ *(Pten*^*d/d*^*Mig-6*^*d/d*^) mice developed EC characterized by neoplastic endometrial glands invading through the myometrium at 1 month of age [31]. In order to examine the association between the levels of ARG1 and EC progression, we performed immunohistochemistry (IHC) analyses for ARG1 in the uteri of *Pten*^*d/d*^ and *Pten*^*d/d*^*Mig-6*^*d/d*^ mice. At 1 month of age, ARG1 proteins were weakly expressed in some endometrial hyperplasia of *Pten*^*d/d*^ mice, whereas these proteins were remarkably strong in *Pten*^*d/d*^*Mig-6*^*d/d*^ mice (Fig. 1 A). Our semi-quantitative analysis revealed a significant (p = 0.0005) 2.4-fold increase in ARG1 expression *Pten*^*d/d*^*Mig-6*^*d/d*^ mice (H-score = 210.7 ± 15.4) compared to *Pten*^*d/d*^ *mice* (H-score = 87.5 ± 15.9) (Fig. 1B). At 2 months of age, the levels of ARG1 proteins were also stronger in EC of *Pten*^*d/d*^*Mig-6*^*d/d*^ mice compared with *Pten*^*d/d*^ mice (Fig. 1C). Invasion of EC into myometrium was observed in both *Pten*^*d/d*^*Mig-6*^*d/d*^ and *Pten*^*d/d*^ mice. Importantly, ARG1 levels in EC of myometrium in *Pten*^*d/d*^*Mig-6*^*d/d*^ (H-score = 269.6 ± 12.6) had a significant (p = 0.0005) 1.7-fold increase compared to *Pten*^*d/d*^ mice (H-score = 184.5 ± 15.0) (Fig. 1D).

### Overexpression of Mig-6 suppresses ARG1 protein expression in Ren deficient EC.

**Figure 2:**
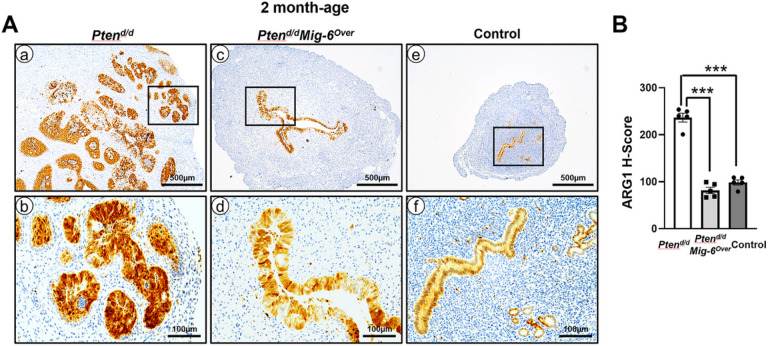
The expression of ARG1 is significantly reduced in *Pten*^*d/d*^*Mig-6*^*Over*^ at 2 months of age. (**A**) Immunohistochemical analysis of ARG1 in the uteri of *Pten*^*d/d*^*, Pten*^*d/d*^*Mig-6*^*Over*^and control mice. (**B**) Quantification of ARG1 positive cells in the uteri of *Pten*^*d/d,*^*Pten*^*d/d*^*Mig-6*^*Over*^and control mice. The results represent the mean ± SEM. ***p < 0.001.

*MIG-6* is known as a tumor-suppressor gene in EC, and overexpression of *Mig-6* suppresses EC progression in *Pten*^*d/d*^ mice [33], Next, we examined if *Mig-6* overexpression reduces the expression of *ARG1* in *Pten*^*d/d*^ mice. Our immunohistochemistry analyses showed that ARG1 proteins were strongly expressed in endometrial hyperplasia and EC that has invaded into the myometrium of *Pten*^*d/d*^ uteri at 2 months of age (Fig. 2A a and b). Interestingly, ARG1 expression was weakly detected in the endometrial epithelium of *Pten*^*d/d*^*Mig-6*^*over*^ uteri (Fig. 2A a and d). In addition, wild type control mice revealed weak ARG1 expression in luminal and glandular epithelial cells. Furthermore, our H-score analysis showed that the levels of ARG1 had a significant (p< 0.0001) 2.9-fold reduction in *Pten*^*d/d*^*Mig-6*^*over*^ mice (H-score = 81.7 ± 6.7) compared to *Pten*^*d/d*^ mice (H-score = 236.5 ± 9.4) (Fig. 2B). Flowever, the levels of ARG1 protein were not different between *Pten*^*d/d*^*Mig-6*^*over*^ and control (H-score = 98.5 ± 5.4) mice (Fig. 2B).

**Figure 3:**
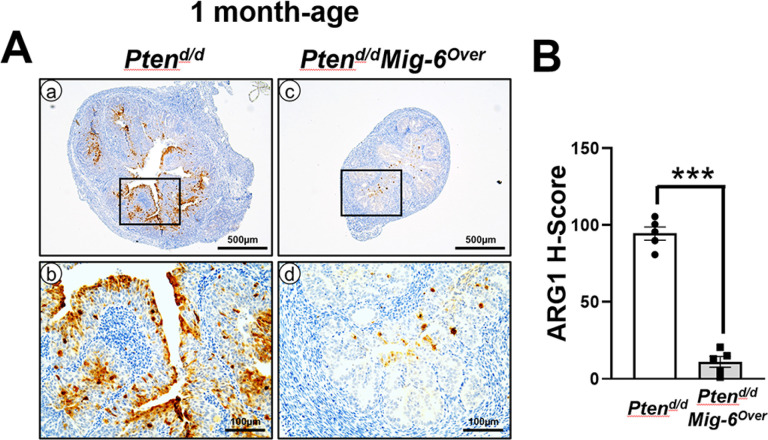
The expression of ARG1 is decreased in *Pten*^*d/d*^*Mig-6*^*Over*^ at 1 month of age. (**A**) Immunohistochemical analysis of ARG1 in the uteri of *Pten*^*d/d*^*, Pten*^*d/d*^*Mig-6*^*Over*^and control mice. (**B**) Quantification of ARG1 positive cells in the uteri of *Pten*^*d/d*^*, Pten*^*d/d*^*Mig-6*^*Over*^and control mice. The results represent the mean ± SEM. ***p < 0.001.

Next, we examined the expression of ARG1 in endometrial hyperplasia of *Pten*^*d/d*^and *Pten*^*d/d*^*Mig-6*^*over*^ mice at 1 month of age. While ARG1 positive cells were strongly expressed in endometrial hyperplasia of *Pten*^*d/d*^ mice, *Pten*^*d/d*^*Mig-6*^*over*^ mice revealed few ARG1 positive cells in endometrial hyperplasia (Fig. 3A). Our H-score analysis demonstrated significant 8.5-fold increase of ARG1 levels (p < 0.0001) in *Pten*^*d/d*^ mice (H-score = 94.5 ± 4.3) compared to *Pten*^*d/d*^*Mig-6*^*over*^ (H-score = 11.1 ±3.5) mice (Fig. 3B).

### Arg1 expression is associated with EC development.

**Figure 4:**
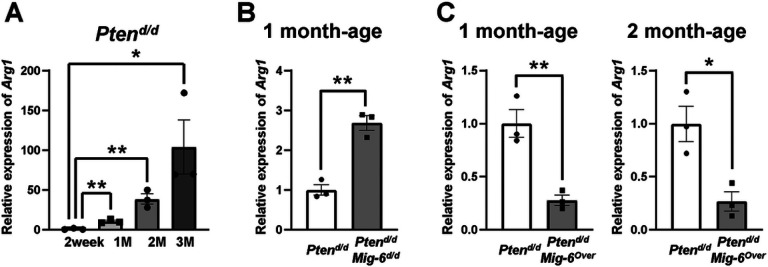
The mRNA expression of *Arg1* is increased during tumorigenesis of *Pten*^*d/d*^ and *Pten*^*d/d*^*Mig-6*^*d/d*^ mice. (**A**) Real-time RT-PCR analysis of *Arg1* was performed on uteri of *Pten*^*d/d*^ mice at 2 weeks, 1 month (1M), 2 months (2M) and 3 months (3M) of age. (**B**) The mRNA expression of *Arg1* in the uteri of *Pten*^*d/d*^ and *Pten*^*d/d*^*Mig-6*^*d/d*^ mice at 1 month of age. (**C**) The mRNA expression of *Arg1* in the uteri of *Pten*^*d/d*^ and *Pten*^*d/d*^*Mig-6*^*Over*^mice at 1 month and 2 months of age. The results represent the mean ± SEM. *p < 0.05, **p < 0.01.

To determine whether the expression of *Arg1* gene is correlated to the development and progression of EC, we examined the mRNA expression of *Arg1* in the *Pten*^*d/d*^ mice at 2 weeks, 1 month, 2 months and 3 months of age. As shown in Fig. 4A, mRNA levels of *Arg1* were significantly increased 10.6-fold (p =0.003), 38.5-fold (p = 0.0044), and 103.8-fold (p = 0.0394) in uteri of *Pten*^*d/d*^ mice at 1 (10.6 ± 1.22), 2 (38.5 ± 5.58), and 3 (103.8 ± 29.53) months of age compared to uteri of *Pten*^*d/d*^ mice at 2 weeks (1.0 ± 0.42) of age, respectively. Our results demonstrate that gradual increase of *Arg1* expression is correlated to progression of EC with *Pten* deficiency.

Next, we examined mRNA levels of *Arg1* in endometrial hyperplasia of *Pten*^*d/d*^*, Pten*^*d/d*^*Mig-6*^*d/d*^ and *Pten*^*d/d*^*Mig-6*^*over*^ mice at 1 month of age. The expression levels of *Arg1* mRNA had a significant (p =0.0017) 2.7-fold increase in *Pten*^*d/d*^*Mig-6*^*d/d*^ mice (2.7 ± 0.16) compared to *Pten*^*d/d*^ mice (1.0 ± 0.12) at 1 month of age (Fig. 4B). Furthermore, the expression of *Arg1* mRNA levels in the of *Pten*^*d/d*^*Mig-6*^*over*^ mice at 1 month (0.28 ± 0.04) and 2 months (0.27 ± 0.08) of age were significantly decreased compared to *Pten*^*d/d*^ mice at 1 month (1.0 ± 0.12; p = 0.0072) and 2 months (1.0 ± 0.15; p = 0.0184) of age, respectively (Fig. 4C). Overall, our results of IFHC and RT-qPCR reveal that ARG1 expression is correlated with progression of EC.

### 2.5 ARG1 as a potential prognostic marker for EC

To evaluate whether the expression of ARG1 is a potential prognostic and predictive marker in EC, *Pten*^*d/d*^*, Pten*^*d/d*^*Mig-6*^*d/d*^ and *Pten*^*d/d*^*Mig-6*^*over*^ mice were sacrificed at 2 weeks of age. While ratios of uterine weight to body weight between *Pten*^*d/d*^ and *Pten*^*d/d*^*Mig-6*^*d/d*^ mice were not different [27], ratios of uterine weight to body weight were significantly reduced by a 2.1-fold change (p < 0.001) in *Pten*^*d/d*^*Mig-6*^*over*^ mice (2.2 ± 0.08) compared to *Pten*^*d/d*^ mice (4.6 ± 0.2) at 2 weeks of age (Fig. 4D). Flowever, histological analysis demonstrated that the uteri of *Pten*^*d/d*^*, Pten*^*d/d*^*Mig-6*^*d/d*^ [27] and *Pten*^*d/d*^*Mig-6*^*over*^ mice exhibited same endometrial hyperplasia (Fig. 4E).

ARG1 protein was rarely detected in endometrial hyperplasia of *Pten*^*d/d*^and *Pten*^*d/d*^*Mig-6*^*d/d*^ mice at 2 weeks of age (Suppl. Figure 1). Flowever, we could not detect ARG1 staining wild type control mice. Importantly, the mRNA expression of *Arg1* in the uteri of *Pten*^*d/d*^*Mig-6*^*d/d*^ mice (4.6 ± 0.73) was significantly 4.6- and 57.2-folds increased compared to *Pten*^*d/d*^ (1.0 ± 0.42) and *Pten*^*d/d*^*Mig-6*^*over*^ mice (0.08 ± 0.01) at 2 weeks of age (p = 0.009 and p = 0.003, respectively) (Fig. 4F). These results suggest that ARG1 is a potential prognostic and predictive marker for EC with *PTEN* deficiency.

## Discussion

Prognosis for early-stage and low grade EC is generally favorable: 5-year survival is 69–88% for FIGO (International Federation of Gynecology and Obstetrics) stage I–II disease [4], Flowever, the prognosis for metastatic and recurrent EC is poor and remains incurable with limited effective treatment options [34, 35], Furthermore, despite successful surgery (hysterectomy), 10–15% of EC tumors recur within 5 years with low survival rates [3], Identification of patients at high risk of metastasis and recurrence could aid in early treatment involving adjuvant treatment or extensive surgical staging. However, it has been challenging to determine which EC patients are at highest risk for distant metastasis and recurrence and who therefore would benefit most from adjuvant treatment or more extensive surgical staging. To identify biomarkers associated with a poor prognosis in EC, we evaluated the relationship between ARG1 levels and cancer progression using our preclinical mouse models including *Pten*^*d/d*^*, Pten*^*d/d*^*Mig-6*^*d/d*^ and *Pten*^*d/d*^*Mig-6*^*over*^ mice.

Lack of realistic models has stymied research on EC metastasis and recurrence for years. We have developed mouse models that implicate concurrent *Pten* and *Mig-6* deficiency in metastatic and recurrent EC [27], While loss of *Pten* is not sufficient for distant metastasis, mice will develop distant metastasis when *Mig-6* and *Pten* are both ablated [27]. We employed preclinical animal models that closely resemble human EC with distant metastasis [23, 27, 36], Our metastatic EC mouse models are low cost and produce EC similar to the EC seen in women. Similarities between our mouse model and human metastatic and recurrent EC include: 1) *PTEN* deficiency; 2) EC tumorigenesis; and 3) pathophysiology of distant metastasis. The mice are vital in the identification of drug candidates with potential to treat EC metastasis and recurrence in humans and will enable further ground breaking studies that aim to understand the underline pathophysiology of EC recurrence and metastasis.

While downstream mechanisms of PTEN inactivation and AKT/mTOR activation are well known in EC [37–39], pathways for suppressing AKT activation in EC are poorly understood. Our discovery that MIG-6 is a negative regulator of AKT opens a new avenue of research to identify prognostic biomarkers and unravel mechanisms of metastasis in EC. Our immunostaining and RT-qPCR revealed a significant increase in ARG1 expression in the endometrial hyperplasia of mice deficient in *Mig-6* and *Pten,* two tumor suppressor genes. Our data suggests that ARG1 is a promising prognostic and predictive biomarker for metastatic and recurrent EC with PTEN deficiency. Further steps involve the evaluation of human subjects with EC, especially those with early-stage EC.

## Figures and Tables

**Figure 5:**
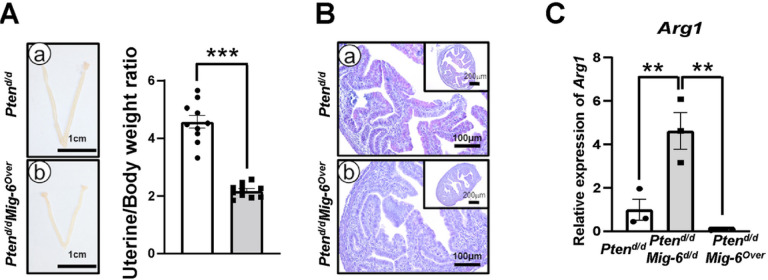
The mRNA expression of *Arg1* is a potential prognostic marker EC with PTEN deficiency. (**A**) Uterus anatomy representative images and uterus/body weight ratio demonstrate a significant reduction of uterine weight in *Pten*^*d/d*^*Mig-6*^*Over*^mice at 2 weeks of age compared to *Pten*^*d/d*^. (**B**) Hematoxylin and eosin (H&E) staining in *Pten*^*d/d*^ and *Pten*^*d/d*^*Mig-6*^*Over*^ mice at 2 weeks of age. Arrowheads indicate embryos. (**C**) The mRNA expression of *Arg1* in the uteri of *Pten*^*d/d*^*, Pten*^*d/d*^*Mig-6*^*d/d*^ and *Pten*^*d/d*^*Mig-6*^*Over*^ mice at 2 weeks of age. The results represent the mean ± SEM. *p < 0.05, **p < 0.01.

## Data Availability

All datasets presented in this study are included in the article. All data is real and guarantee the validity of experimental results.

## Data Availability

The data underlying this article are available in the article and in its online supplementary material.
